# Long-term survival in pseudo-Meigs’ syndrome caused by ovarian metastases from colon cancer

**DOI:** 10.1186/s12957-016-1040-0

**Published:** 2016-11-14

**Authors:** Yosuke Tajima, Hitoshi Kameyama, Saki Yamada, Ryoma Yagi, Masato Nakano, Masayuki Nagahashi, Yoshifumi Shimada, Jun Sakata, Takashi Kobayashi, Hajime Umezu, Toshifumi Wakai

**Affiliations:** 1 Division of Digestive and General Surgery, Niigata University Graduate School of Medical and Dental Sciences, 1-757 Asahimachi-dori, Chuo-ku, Niigata, 951-8510 Japan; 2 Division of Pathology, Niigata University Graduate School of Medical and Dental Sciences, 1-757 Asahimachi-dori, Chuo-ku, Niigata, Japan

**Keywords:** Pseudo-Meigs’ syndrome, Meigs’ syndrome, Colorectal cancer, Ovarian metastasis, Long-term survival

## Abstract

**Background:**

Meigs’ syndrome is defined as the co-existence of benign ovarian fibroma or fibroma-like tumor, ascites, and pleural effusion. In contrast, pseudo-Meigs’ syndrome is defined as the co-existence of other ovarian or pelvic tumors, ascites, and pleural effusion. In Meigs’ and pseudo-Meigs’ syndromes, ascites and pleural effusion resolve promptly after the complete resection of the ovarian or pelvic tumor(s). Secondary ovarian tumors from colorectal gastrointestinal metastases rarely cause pseudo-Meigs’ syndrome; only 11 cases of pseudo-Meigs’ syndrome secondary to colorectal cancers have been reported in the literature. Therefore, the prognosis and etiology of pseudo-Meigs’ syndrome caused by ovarian metastasis from colorectal cancers remain unclear.

**Case presentation:**

We report here a rare case of pseudo-Meigs’ syndrome caused by ovarian metastases from sigmoid colon cancer with long-term survival. A 47-year-old woman presented with abdominal distention of 1-month duration. She developed acute dyspnea 2 weeks after the initial presentation. Colonoscopy and computed tomography revealed sigmoid colon cancer with an ovarian metastasis, along with massive ascites and bilateral pleural effusion. Emergency operation, including bilateral oophorectomy and sigmoidectomy, was performed. Subsequently, ascites and bilateral pleural effusion resolved rapidly. Curative hepatic resection was performed for liver metastases 29 months after the first operation, and as of this writing, the patient is alive with no evidence of a disease 78 months after the first operation. In general, colorectal cancer with ovarian metastasis is hard to cure, and long-term survival in patients with colorectal cancer with pseudo-Meigs’ syndrome is rare. Our experience suggests that curative resection for pseudo-Meigs’ syndrome caused by ovarian metastasis from colorectal cancer may offer long-term survival.

**Conclusions:**

Our experience suggests that pseudo-Meigs’ syndrome can occur in a patient with colorectal cancer after metastasis to the ovaries, causing massive ascites and pleural effusion. Aggressive treatment, including R0 resection, for this disease if allowed by the patient’s general condition may offer long-term survival.

## Background

Meigs’ syndrome is defined as the co-existence of benign ovarian fibroma or fibroma-like tumor, ascites, and pleural effusion [1]. The features of this disease were first described by Meigs and Cass in 1937 [2]. In contrast, pseudo-Meigs’ syndrome is defined as the co-existence of other ovarian or pelvic tumors, ascites, and pleural effusion [3]. In Meigs’ and pseudo-Meigs’ syndromes, ascites and pleural effusion resolve promptly after the complete resection of the ovarian or pelvic tumor(s) [1].

Secondary ovarian tumors from colorectal gastrointestinal metastases rarely cause pseudo-Meigs’ syndrome; only 11 cases of pseudo-Meigs’ syndrome secondary to colorectal cancers have been reported in the literature (Table 1) [4–10]. Therefore, the prognosis and etiology of pseudo-Meigs’ syndrome caused by ovarian metastasis from colorectal cancers remain unclear. Furthermore, differentiating between pseudo-Meigs’ syndrome and carcinomatous peritonitis/pleuritis as well as between ovarian metastasis and primary ovarian cancer is often difficult.

**Table 1:** Summary of reported cases of pseudo-Meigs’ syndrome resulting from ovarian metastasis of colorectal cancer

Reference	Age	Onset of syndrome	Synchronous metastasis	Diameter of ovarian tumor (cm)	Site of ovarian tumor	Site of pleural effusion	Curative resection	Long-term outcome
Nagakura et al. [4]	35	Metachronous	None	15	Unilateral	Right	Yes	108 months, NED
Nagakura et al. [4]	40	Synchronous	None	ND	Bilateral	Right	Yes	ND
Nagakura et al. [4]	39	Synchronous	None	24	Unilateral	Bilateral	Yes	12 months, NED
Nagakura et al. [4]	75	Synchronous	Peritoneum	21	Unilateral	Right	No	ND
Nagakura et al. [4]	53	Synchronous	None	18	Bilateral	Right	Yes	52 months, AWD
Feldman et al. [5]	49	Metachronous	None	13	Unilateral	Left	Yes	6 months, NED
Ohsawa et al. [6]	41	Synchronous	Peritoneum	16	Bilateral	Bilateral	ND	9 months, dead
Rubinstein et al. [7]	61	Synchronous	None	13	Bilateral	Bilateral	Yes	ND
Okuchi et al. [8]	42	Synchronous	Liver, lung	11.5	Unilateral	Right	No	12 months, dead
Maeda et al. [9]	58	Synchronous	Peritoneum	15	Bilateral	Right	Yes	10 months, NED
Saito et al. [10]	44	Synchronous	ND	22.8	ND	Bilateral	ND	ND
Present case	47	Synchronous	Peritoneum	18	Bilateral	Bilateral	Yes	78 months, NED

*ND* not documented, *NED* alive with no evidence of disease, *AWD* alive with disease

We report here a rare case of pseudo-Meigs’ syndrome caused by ovarian metastasis from sigmoid colon cancer, with the patient showing long-term survival following complete tumor resection.

## Case presentation

**Fig. 1:**
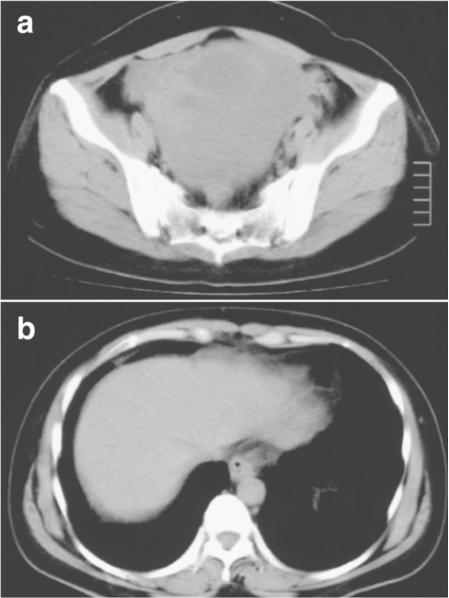
Computed tomography demonstrated a large, round mass with a maximum diameter of 15 cm in the pelvic cavity (**a**) and no acites or pleural effusion (**b**)﻿

**Fig. 2:**
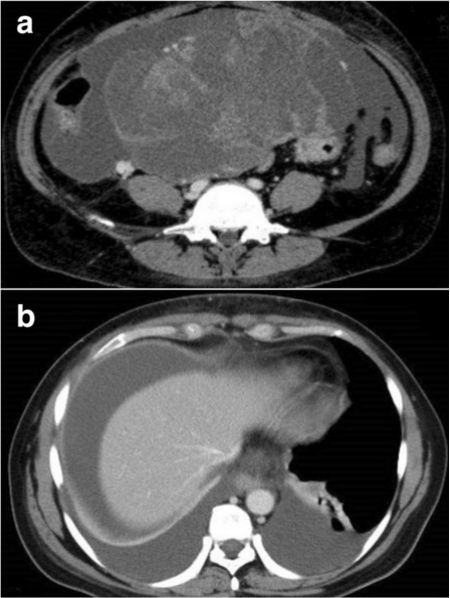
The second computed tomographic scan, obtained 2 weeks after the initial scan, demonstrated massive ascites (**a**) and bilateral pleural effusion (**b**)

**Fig. 3:**
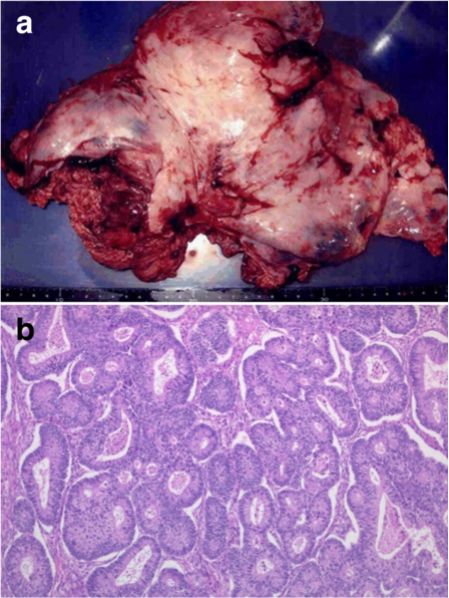
Gross pathology. **a** Macroscopic findings showed a right ovarian tumor with cystic and solid portion measuring 18 × 15 × 11 cm. **b** Microscopic findings showed moderately differentiated adenocarcinoma in both ovaries on hematoxylin and eosin staining (magnification ×200)

**Fig. 4:**
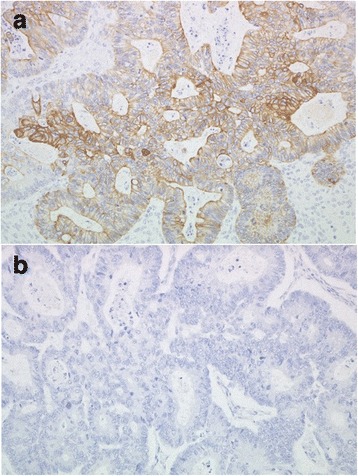
Immunohistochemical findings. Immunohistochemical staining of the ovarian tumor cells showed a positive reaction for cytokeratin 20 (**a**) (magnification ×200) but no reaction for cytokeratin 7 (**b**) (magnification ×200)

A 47-year-old woman presented to the hospital with a 1-month history of abdominal distention. Physical examination revealed a huge mass in the lower abdomen. The peripheral blood test showed elevated levels of carcinoembryonic antigen (CEA, 335.2 ng/mL [normal, <5 ng/mL]) and carbohydrate antigen (CA) 125 (219 U/mL [normal, <45 U/mL]) but a normal level of CA19-9 (9 U/mL [normal, <37 U/mL]). Computed tomography (CT) demonstrated a large, round mass with a maximum diameter of 15 cm in the pelvic cavity without the presence of ascites or pleural effusion (Fig. 1a, b). Colonoscopy identified an elevated lesion with severe stenosis in the sigmoid colon, and histopathological examination of biopsy specimens from the tumor showed moderately differentiated adenocarcinoma. Although we scheduled an early operation, the patient developed acute dyspnea and general edema 2 weeks after the first CT scan. The second CT scan examination demonstrated massive bilateral pleural effusion with atelectasis and ascites (Fig. 2a, b). Thoracic drainage and laparotomy were emergently performed. Macroscopically, the tumor in the sigmoid colon had invaded the serosa, and the huge pelvic mass was found to contain a right ovarian tumor. Several small nodules of peritoneal dissemination were distributed over the greater omentum. Perioperatively, 3800 mL of serous ascitic fluid was drained. Cytodiagnosis of the fluid drained from the ascites and pleural effusion revealed no tumor cells. Bilateral oophorectomy, total hysterectomy, omentectomy, and sigmoidectomy with regional node dissection were performed (Fig. 3a). Histopathological examination of the resected specimens showed moderately differentiated adenocarcinoma in the tumors of both the ovaries and the sigmoid colon (Fig. 3b). The dissected paracolic nodes showed malignant cells. Immunohistochemically, tumor cells from the ovaries and the colon both showed positive expression of cytokeratin 20 (CK20) but no expression of cytokeratin 7 (CK7), confirming that the ovarian tumors were metastases from primary colon cancer (Fig. 4a, b). The postoperative course was uneventful, and both pleural effusion and ascites rapidly resolved. Postoperatively, a regimen of 5-fluorouracil (5-FU), leucovorin, and oxaliplatin (FOLFOX) was administered every 2 weeks for 5 months. At 29 months after the first operation, the patient required curative hepatic resection for liver metastases. At 78 months after the first operation, the patient remains alive with no evidence of a disease.

### Discussion

Our experience in this case indicated that R0 resection may offer a good prognosis and outcomes for patients with colorectal cancer and pseudo-Meigs’ syndrome caused by ovarian metastases. Among such cases reported thus far, 8 of the 12 reported patients—including ours—underwent R0 resection, and some showed long-term survival [4–10]. In contrast, long-term survival was not observed in any of the patients that did not undergo R0 resection [4–10]. In our case, bilateral oophorectomy, omentectomy, and sigmoidectomy with regional lymph node dissection were performed. Subsequently, hepatic resection was performed for metachronous liver metastases, and at 78 months after the first operation, the patient remains alive with no evidence of a disease. In general, R0 resection can be the first choice in the treatment of colorectal cancer with ovarian metastasis [11]. Therefore, we suggest that R0 resection should be performed for colorectal cancer with pseudo-Meigs’ syndrome caused by ovarian metastasis.

The differential diagnosis between pseudo-Meigs’ syndrome due to ovarian metastasis from colorectal cancer and carcinomatous pleuritis/peritonitis is important, because R0 resection can be indicated for the former but not for the latter. However, a confirmed diagnosis based on clinical or imaging findings is rather difficult. Cytodiagnosis of the fluid drained from pleural effusion and ascites is useful in the diagnosis; negative findings tend to imply pseudo-Meigs’ syndrome, whereas positive findings imply carcinomatous pleuritis or peritonitis. In our case, the intraoperative cytodiagnosis of the ascitic and pleural effusion fluids showed negative findings. Furthermore, the size and number of the disseminated peritoneal nodules were so small that they did not seem to be the cause of the massive ascites. We therefore diagnosed pseudo-Meigs’ syndrome and performed R0 resection.

The differential diagnosis between metastatic ovarian cancer and primary ovarian cancer is also important, because chemotherapy regimens for colorectal cancer differ considerably from those of ovarian cancer. The serum level of CA125 is likely to be elevated in both primary and metastatic ovarian cancer [12]. Immunohistochemical staining of the resected ovarian tumor is useful for diagnosis; the positive expression of CK20 and no expression of CK7 suggest metastases from colorectal cancer, whereas the positive expression of CK7 and no expression of CK20 indicate primary ovarian cancer [13]. In our patient, we diagnosed the ovarian tumors as metastases from colon cancer based on immunohistochemical staining. Accordingly, we administered FOLFOX as an adjuvant chemotherapy.

The details of the mechanism underlying the occurrence of ascites and pleural effusion in pseudo-Meigs’ syndrome remain unclear. One hypothesis is that ascites is caused by a disparity between the arterial supply to the ovarian tumor and its venous and lymphatic drainage [1, 14], and the ascites finds its way to the thoracic cavity through intercellular gaps via aortic, esophageal, and vena caval pathways and a number of smaller avenues [15, 16]. Dye test results have shown that the pleural effusion is likely to originate from the peritoneal fluid via transdiaphragmatic transport [17]. This hypothesis supports the fact that both ascites and pleural effusion contain no malignant cells and disappear promptly after the complete resection of the ovarian or pelvic tumors in pseudo-Meigs’ syndrome.

## Conclusions

Our experience suggests that pseudo-Meigs’ syndrome can occur in a patient with colorectal cancer after metastasis to the ovaries, causing massive ascites and pleural effusion. Aggressive treatment, including R0 resection, for this disease if allowed by the patient’s general condition may offer long-term survival.

## Abbreviations

5-FU: 5-Fluorouracil
CA: Carbohydrate antigen
CEA: Carcinoembryonic antigen
CK: Cytokeratin
CT: Computed tomography
FOLFOX: A regimen of 5-fluorouracil, leucovorin, and oxaliplatin
